# Global MicroRNA Profiling of the Mouse Ventricles during Development of Severe Hypertrophic Cardiomyopathy and Heart Failure

**DOI:** 10.1371/journal.pone.0044744

**Published:** 2012-09-14

**Authors:** Richard D. Bagnall, Tatiana Tsoutsman, Rhian E. Shephard, William Ritchie, Christopher Semsarian

**Affiliations:** 1 Agnes Ginges Centre for Molecular Cardiology, Centenary Institute, Sydney, New South Wales, Australia; 2 Sydney Medical School, University of Sydney, New South Wales, Australia; 3 Department of Bioinformatics, Centenary Institute, Royal Prince Alfred Hospital, Sydney, New South Wales, Australia; 4 Department of Cardiology, Royal Prince Alfred Hospital, Sydney, New South Wales, Australia; Bristol Heart Institute, University of Bristol, United Kingdom

## Abstract

MicroRNAs (miRNAs) regulate post-transcriptional gene expression during development and disease. We have determined the miRNA expression levels of early- and end-stage hypertrophic cardiomyopathy (HCM) in a severe, transgenic mouse model of the disease. Five miRNAs were differentially expressed at an early stage of HCM development. Time-course analysis revealed that decreased expression of miR-1 and miR-133a commences at a pre-disease stage, and precedes upregulation of target genes causal of cardiac hypertrophy and extracellular matrix remodelling, suggesting a role for miR-1 and miR-133a in early disease development. At end-stage HCM, 16 miRNA are dysregulated to form an expression profile resembling that of other forms of cardiac hypertrophy, suggesting common responses. Analysis of the mRNA transcriptome revealed that miRNAs potentially target 15.7% upregulated and 4.8% downregulated mRNAs at end-stage HCM, and regulate mRNAs associated with cardiac hypertrophy and electrophysiology, calcium signalling, fibrosis, and the TGF-β signalling pathway. Collectively, these results define the miRNA expression signatures during development and progression of severe HCM and highlight critical miRNA regulated gene networks that are involved in disease pathogenesis.

## Introduction

MicroRNAs (miRNAs) are non-coding RNA molecules of ∼22 nucleotides that regulate post-transcriptional gene expression, and over 1900 are known to exist in humans [1]. miRNAs are transcribed as precursor transcripts, which fold to form miRNA-5p:miRNA-3p stem-loop duplexes [2]. The precursor transcripts are cleaved in the nucleus by Drosha into ∼70 nucleotide pre-miRNAs and then transported to the cytoplasm for further processing by Dicer before one strand of the stem-loop duplex is incorporated into the RNA-induced silencing complex [2]. miRNAs can also derive from introns of protein coding genes that are spliced and debranched into pre-miRNA hairpin mimics by a Drosha independent pathway involving the spliceosome, before being cleaved by Dicer into functional miRNAs [3]. miRNAs negatively regulate gene expression through binding of nucleotide positions 2–8 from the 5′ end of the miRNA, or seed sequence, to complementary target sequences in the 3′ untranslated region of target messenger RNAs (mRNAs) [4]. Target recognition is also influenced by the secondary structure of regions surrounding the target, and the extent of seed sequence complementarity influences whether the miRNA and mRNA interaction results in translation repression or degradation of the bound mRNA. Some miRNAs group into families sharing an identical seed sequence and similar mature sequence, and they likely target the same mRNAs to different extents [1]. miRNAs display a characteristic spatial, temporal and tissue specific expression profile during development and disease, and impact on the expression of many genes and functionally related gene networks [2], [5].

Changes in cardiac miRNA expression levels have been characterised in mice following cardiac stress and development of hypertrophy resulting from thoracic aortic banding (TAB), myocardial infarction, drug treatment and transgene expression [6], [7], [8], [9], [10], and in human end-stage heart failure [6], [11], [12]. While there are overlaps in the miRNA expression profiles resulting from these different cardiac stresses, suggesting some common responses, there are also miRNAs that show differential expression unique to each cardiac stress. Since the seed sequence generally differs between miRNA families, each can regulate a different series of mRNAs. This has lead to the notion that cardiac miRNA expression profiles represent novel and sensitive signatures of disease, and that the target mRNAs highlight networks of genes with a central role in cardiovascular disease.

**Figure 1:**
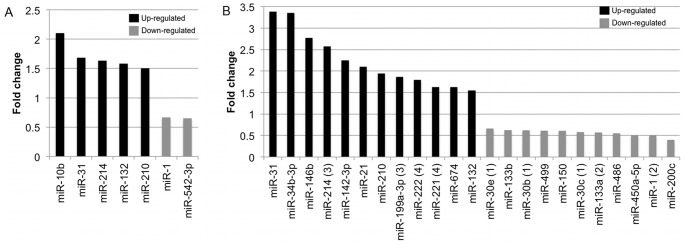
Differentially expressed miRNAs during development of HCM. Fold change is shown for DBL mice; **A**) age 10 days, and **b**) age 16 days v age matched NTG mice. (1) miRNAs are part of the same family, (2) and (3) part of the same transcription unit, or (4) clustered.

**Table 1:** Validated miRNAs differentially expressed in DBL mice.

	Pre-disease(5 day old)	Early disease(10 day old)	Established disease(14 day old)	End disease(16 day old)
**Up Regulated miRNAs**		mir-21	mir-21	mir-21
		mir-331	mir-31	mir-31
		mir-132	mir-34b-3p	mir-34b-3p
		mir-214	mir-132	mir-132
			mir-142	mir-142
			mir-214	mir-214
			mir-222	mir-222
	mir-1	mir-1	mir-1	mir-1
**Down Regulated miRNAs**	mir-133a		mir-30b-5p	mir-30-5p
			mir-30c	mir-30c
			mir-30e	mir-30e
			mir-133a	mir-133a
			mir-133b	mir-133b
			mir-150	mir-150
			mir-486-5p	mir-486-5p
				mir-499-5p

MicroRNAs significantly upregulated (top panel) or downregulated (bottom panel) (*P*<0.05).

Hypertrophic cardiomyopathy (HCM) is a primary disorder of the myocardium characterised by unexplained left ventricular hypertrophy, fetal gene upregulation and defining histological features of myocyte hypertrophy and disarray, and interstitial myocardial fibrosis [13]. HCM is caused by autosomal dominant mutations in over 13 genes that primarily encoding proteins of the sarcomere [14]. Up to 5% of HCM cases carry two independent mutations that lead to more severe clinical disease, including greater left ventricular hypertrophy and a higher incidence of sudden cardiac death events [15], [16], [17]. Moreover, patients with double mutations are significantly younger at diagnosis and more commonly present with childhood-onset hypertrophy [15], [18], [19]. While the genetic causes of HCM are largely known, it is less clear how individual sarcomere gene mutations alter intracellular signalling, leading to cardiac remodelling and hypertrophy [14]. We have recently developed a double-mutant mouse model of HCM (DBL) by crossbreeding mice with the HCM-causing mutations Gly203Ser in cardiac troponin I (TnI-203) and Arg403Gln in α-myosin heavy chain (MHC-403) [20]. DBL mice develop a severe cardiac phenotype with a significantly increased ratio of heart weight to body weight, marked interstitial myocardial cardiac fibrosis, conduction system abnormalities, heart failure and death by age 21 days. The DBL mouse model represents an important molecular tool to facilitate an improved understanding of the pathogenesis of HCM, and replicates the subgroup of human HCM patients who have severe heart failure requiring aggressive treatment, including transplantation [21].

The present study sought to determine the global miRNA expression profiles of ventricles during early and end-stage HCM in the DBL mouse model. Furthermore, global gene expression levels of non-transgenic (NTG) and DBL mice were compared to determine the potential impact of miRNA regulation on mRNA transcript levels and identify miRNA-regulated genes to gain a better understanding of signalling pathways underlying the development of cardiac hypertrophy and extracellular matrix remodelling in HCM.

**Figure 2:**
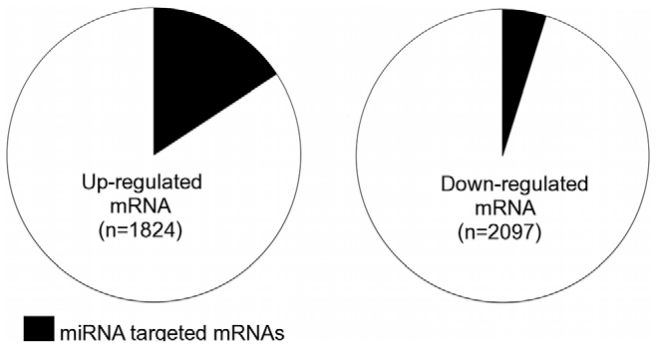
Potential impact of miRNA regulation on mRNA transcript levels. mRNAs upregulated (left) and downregulated (right) during late-stage HCM are represented. Proportion of mRNAs with conserved target sites for conversely expressed miRNAs is shown in black.

## Materials and Methods

### 2.1 Transgenic Mouse Model of Severe HCM

The TnI-203/MHC-403 (DBL) mouse model has been previously described in detail [20]. In brief, DBL mice have mutations in both the myosin heavy chain gene (Arg403Gln) and cardiac troponin I gene (Ser203Gln), resulting in severe HCM, heart failure, and premature mortality by age 21 days. Mice were euthanized and ventricles collected from the excised hearts, snap frozen on dry ice and stored at −80°C. The Sydney University Animal Ethics Committee approved all experiments involving mice.

### 2.2 RNA Extraction and Quantification

Total RNA was extracted from ventricles homogenized in Trizol Reagent (Life Technologies, CA, USA) according to the manufacturer’s instructions, and resuspended in DEPC treated water. RNA was quantified (NanoDrop 2000; Thermo Scientific, MA, USA) and diluted to 150 ng/µl; integrity and purity were measured (RNA 6000 Nano assay; Agilent, Waldbronn, Germany) and RNAs with an RNA integrity number >9 were used for global miRNA analysis and validation studies.

**Table 2:** Validated mRNA targets of miRNA regulation.

Gene	Fold Change	Step-up(p-value)	ValidatedTarget miRNA	Reference
CALM1	+1.18	0.00054	1	[25]
CCND2	+1.38	1.90E-06	1	[39]
CTGF	+5.53	2.81E-12	133	[30]
PPARA	−1.60	7.00E-06	21	[40]
THRB	−1.43	6.38E-05	21	[41]
TWF1	+1.29	8.52E-05	1	[42]

### 2.3 Global miRNA Profiling

The expression levels of 335 murine miRNAs from ventricles of male NTG and DBL mice age 10 and 16 days (n = 3 per group) were measured by real-time quantitative PCR (RT-qPCR) using TaqMan Low Density Array (TLDA) cards, in strict accordance with the manufacturer’s instructions. Briefly, 450 ng RNA was reverse transcribed, without pre-amplification, using TaqMan MicroRNA Reverse Transcription Kit and Megaplex RT Primers rodent pool A (Life Technologies). Complementary DNA (cDNA) was amplified using a TaqMan rodent microRNA A Array v2.0 (Life Technologies) with TaqMan Universal PCR Master Mix on an ABI 7900HT Sequence Detection System. Samples showing no amplification or bad passive reference dye values were excluded and comparative threshold (Ct) values were calculated using SDS software v2.4 (Life Technologies), with automatic baseline settings and a threshold of 0.2. Data were imported into DataAssist software version 2.0 (Life Technologies), which calculates normalizing genes based on geNorm calculations [22]. snoRNA135 and snoRNA202 were used as reference genes to normalize the data as they recorded the lowest stability (M) values (0.25). Mean relative quantity (RQ) was calculated and miRNAs differentially expressed between groups were defined as those with >1.5-fold change and *P*<0.05 (two sample, two tailed Student’s *t*-test), and further checked by manual inspection of amplification curves for consistent separation of all samples between groups.

### 2.4 Validation of TaqMan Low Density Arrays

Individual miRNA RT-qPCR assays (Life Technologies) were performed according to the manufacturer’s instructions, in duplicate, on RNA isolated from the ventricles of male NTG and DBL mice aged 5 (pre-disease), 10 (early disease), 14 (established disease) and 16 days (end-stage disease), with 4 samples in each group. Reverse transcription of 10 ng RNA was performed using TaqMan Reverse Transcription Kit and a specific miRNA assay reverse transcription primer. RT-qPCR was performed on a 1∶20 dilution of cDNA using miRNA assays (Life Technologies) and TaqMan Universal PCR Master Mix on an ABI 7900HT Sequence Detection System. snoRNA135 and snoRNA202 were used to calculate the normalisation factor using geNorm (v 3.4) software [22]. Statistical analyses were performed in GraphPad Prism software v5 using a Mann-Whitney unpaired t-test, with *P*<0.05 considered statistically significant.

### 2.5 Gene Expression Profiling

Global gene expression levels were measured using Affymetrix GeneChip® Mouse Gene 1.0 ST Arrays, according to the manufacturer’s specifications, on RNA isolated from the ventricles of male NTG and DBL mice age 5 and 17 days (n = 5–6 per group). Tissue was homogenised (Polytron pt-MR2100, Kinematica AG, Lucerne, Switzerland) and RNA isolated using RNeasy fibrous tissue kit (Qiagen, Hilden, Germany). RNA quality and quantity were assessed using SYBR Green II assay, agarose gel electrophoresis and RNA 6000 Nano assay (Agilent). Quality control of array data was performed using Expression Console (v1.0) and analysed using Partek Genomics Suite (v6.08.0205) software. Probe signals were normalised using RMA background correction, quantile normalisation and log transformation. Statistical analyses were performed using ANOVA, with a false discovery rate step-up *P*<0.005 considered statistically significant.

**Figure 3:**
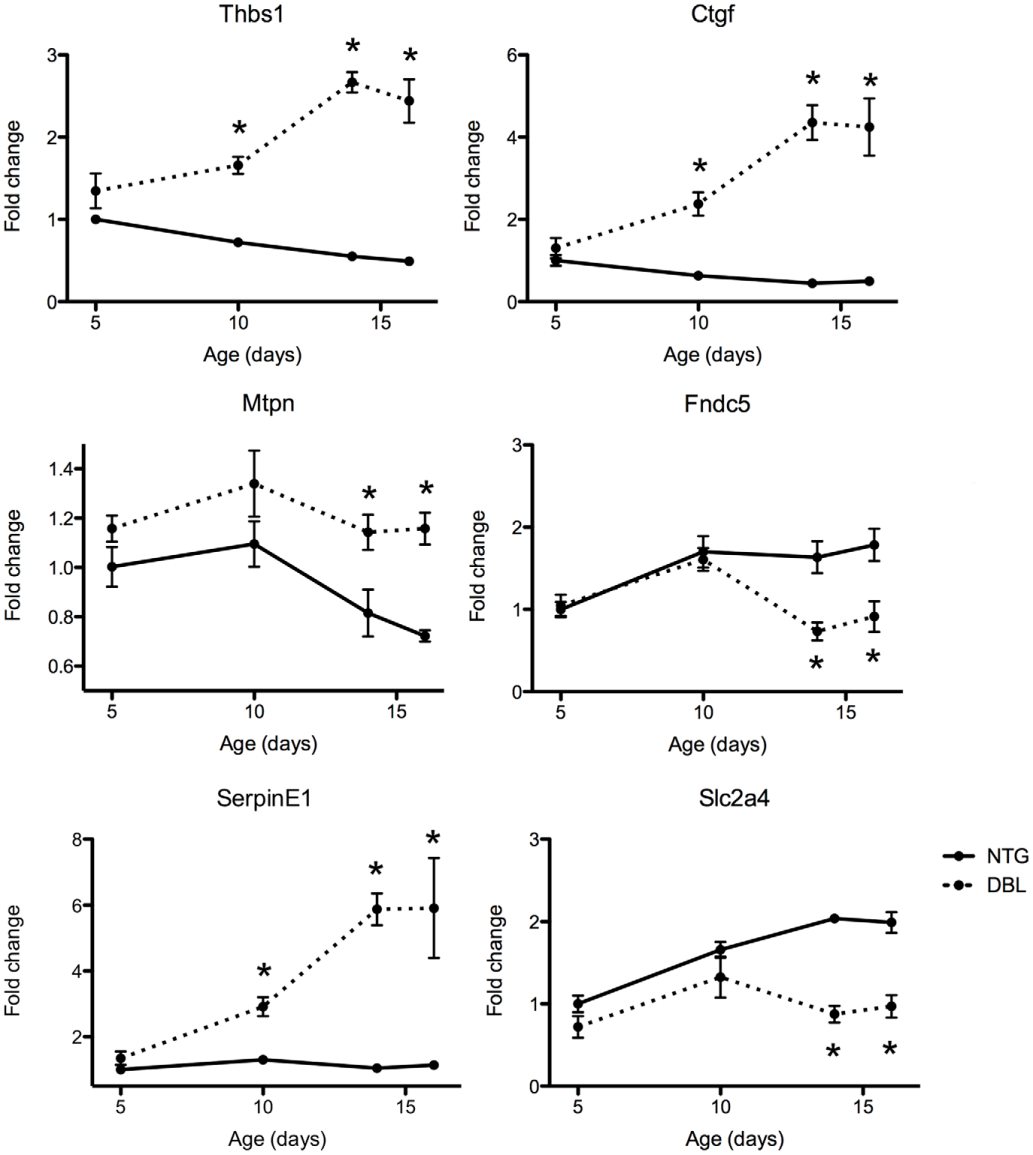
RT-qPCR validation of changes in mRNA expression levels. Extended time course analysis of mRNA expression levels. Fold change is shown compared to NTG mice age 5 days. *P<0.05.

**Table 3:** Cardiac disease-related mRNA targets of differentially expressed miRNA.

Gene	Target miRNA	Role
Myotrophin	1	Hypertrophy
Twinfilin 1	1	Hypertrophy
Insulin-like growth factor 1	30	Hypertrophy
Insulin-like growth factor 1 receptor	30	Hypertrophy
MKL/myocardin-like protein 1	1	Hypertrophy
G protein, alpha inhibiting activity 2	30	Hypertrophy
E2F transcription factor 3	30	Cardiomyocyte proliferation
Fibroblast growth factor receptor 1	133	Cardiomyocyte proliferation
Forkhead box P1	1, 133, 150	Cardiomyocyte proliferation
Cyclin D2	133	Cardiomyocyte proliferation
Collagen I alpha 1	133	Cardiac fibrosis
Connective tissue growth factor	133	Cardiac fibrosis
Epidermal growth factor receptor	133	Cardiac fibrosis
Tissue inhibitor of metalloproteinase 2	30	Cardiac fibrosis
Serpin peptidase inhibitor E	30	Cardiac fibrosis
Thrombospondin 1	1	Cardiac fibrosis
Thrombospondin 1	1	TGF-b signalling pathway
MAD homolog 1	30	TGF-b signalling pathway
MAD homolog 2	486	TGF-b signalling pathway
Activin A receptor 1	30	TGF-b signalling pathway
Calmodulin 1	1, 133	Calcium signalling
Stanniocalcin 1	30	Calcium signalling
Solute carrier 2, member 4	31	Calcium signalling
Solute carrier 8, member 1	1	Cardiac electrophysiology
Sodium channel, voltage-gated, type 4b	31	Cardiac electrophysiology
DnaJ (Hsp40) homolog A2	21	Cardiac electrophysiology
Regulator of G-protein signaling 6	222	Cardiac electrophysiology
AHNAK nucleoprotein	30	Cardiac electrophysiology
Calumenin	30	Cardiac electrophysiology

Target miRNA predicted by TargetScan v6.0 algorithm.

### 2.6 Validation of mRNA Expression Levels

Validation of gene expression levels were performed in duplicate on RNA isolated from the ventricles of four male NTG and DBL mice aged 5 days (pre-disease), 10 days (early disease), 14 days (established disease) and 16 days (end-stage disease). cDNA was prepared using SuperScript® VILO cDNA synthesis kit (Life Technologies) and RT-qPCR was performed using EXPRESS SYBR® GreenER qPCR SuperMix (Life Technologies) on an Mx3000P RT-PCR System, and data analysed using MxPro qPCR software v2.0 (Stratagene, La Jolla, CA, USA). Three reference genes *(β2M, Gapdh* and *βAct*) were used to calculate the normalisation factor using geNorm (v 3.4) software [22]. Statistical analyses were performed in GraphPad Prism software v5 using a Mann-Whitney unpaired *t-*test, with *P*<0.05 considered statistically significant.

### 2.7 Identification of Predicted mRNA Targets of miRNA Regulation

A search was performed for *in-silico* predicted mouse mRNA targets of differentially expressed miRNAs using TargetScan v6.0 algorithm (http://www.targetscan.org/), and limited to genes with conserved target sites.

### 2.8 Gene Ontology Analysis

Statistically over-represented biological annotations (gene terms) were sought from a list of up- or downregulated mRNAs with predicted miRNA target sites, compared to a corresponding background list of all up- or downregulated mRNAs, using DAVID bioinformatics resources [23]. Benjamini-Hochberg globally corrected enrichment *P* values of <0.05 were considered statistically significant.

## Results

### 3.1 Global miRNA Profiling of Ventricles from NTG and DBL Mice

We used TaqMan Low Density Array (TLDA) cards to screen the expression levels of 335 murine miRNAs from ventricles of male NTG and DBL mice, age 10 and 16 days, with 3 mice in each group. We restricted our analysis to miRNAs with a Ct value of <32, as they showed a very high correlation (r = >0.99; Pearson's product moment correlation coefficient) between samples within each group (Figure S1). A total of 177 miRNAs fulfilled these criteria in one or more groups, of which 151 were common amongst all four groups of mice (Table S1). Nine miRNAs with the highest expression levels (average Ct value range 19.6–22.5) were common amongst the four groups of mice despite the differences in age and disease state, and they were miR-133a, miR-126-3p, miR-24, miR-30c, miR-30b, miR-1, miR-16, miR-19b and miR-145 (Table S1).

We compared the TLDA miRNA profiles of NTG and DBL mice at age 10 days to determine the involvement of miRNAs in the development of early-stage HCM. Seven miRNAs showed differential expression (Figure 1a, Figure S2a); miR-1 and miR-542-3p showed decreased expression, whereas miR-132, miR-214, miRNA-31, miR-210 and miR-10b showed increased expression. At age 16 days (end-stage disease), there was decreased expression of 11 miRNAs and increased expression of 12 miRNAs in DBL mice compared to NTG mice (Figure 1b, Figure S2b). Downregulated miRNAs included miR-1 and miR-133a, which are part of the same transcriptional unit, and three miR-30 family members, namely miR-30b, miR-30c and miR-30e. The expression levels of miR-132 and miR-214, which were increased at age 10 days in the DBL mice, remained elevated at age 16 days. miR-199a-3p, which is co-transcribed with miR-214, was also downregulated. miR-31 recorded the highest fold change (3.38x) in DBL mice at age 16 days, followed by miR-34b-3p (3.35x), miR-146b (2.77x), miR-214 (2.57x), miR-142-3p (2.24x) and the cardiac stress responsive miR-21 (2.09x).

### 3.2 Validation of miRNA Expression Levels in NTG and DBL Mice

To validate the TLDA data and extend the time-course of miRNA expression analysis, all of the differentially expressed miRNAs at early and end-stage of HCM were analysed individually at additional time-points using RT-qPCR (Figure S3). Sixteen out of 24 miRs (64%) were validated as having differential expression during the disease course (Table 1). At a pre-disease stage, the co-transcribed miR-1 and miR-133a were significantly lower in DBL mice compared to NTG mice. At 10 days of age, five miRNAs were dysregulated to form an early-stage HCM miRNA expression profile. By 16 days of age, nine miRNAs were significantly lower and 7 miRNAs significantly higher than the age matched NTG mice to form an end-stage HCM miRNA expression profile. In contrast to the TLDA data, nine miRNAs were not validated as having an altered expression level during the disease course (Figure S3C) and collectively they had higher Ct values than the validated miRNAs, denoting lower abundance (*P* = 0.002 Mann Whitney two-tailed t test).

### 3.3 Potential Extent of miRNA Regulation of mRNA Transcript Levels

MicroRNAs can regulate post-transcriptional gene expression by targeting mRNAs for degradation. In order to explore the potential extent of miRNA-directed regulation of mRNA levels, GeneChip Mouse Exon Arrays were used to measure mRNAs differentially expressed during the course of HCM. A total of 1824 upregulated and 2097 downregulated mRNAs (*P*<0.005) were revealed at end-stage HCM compared to a pre-disease stage and these mRNAs could be regulated by the conversely, differentially expressed miRNAs.

At end-stage HCM, 287 out of 1824 (15.7%) upregulated mRNAs and 101 out of 2097 (4.8%) downregulated mRNAs contained conserved predicted target sites for one or more of the conversely expressed end-stage HCM signature miRNAs (Figure 2), and these proportions did not change when considering mRNAs differentially expressed at the *P*<0.001 level. Furthermore, mRNAs targeted by the end-stage HCM signature miRNAs were significantly more abundant among the differentially expressed mRNAs than compared to all mRNAs included on the GeneChip Mouse Exon Array (*P*<2.5^−4^ Pearson's Chi-squared test with Yates’ continuity correction). Of these potential mRNA targets of miRNA regulation, 6 have been directly validated using luciferase assays (Table 2). Analysis of related gene ontology terms did not reveal any overrepresentation of functional categories in the dysregulated mRNAs with predicted miRNA target sites, however, many are known to be involved in processes well studied in heart failure (Table 3). A subset of predicted target mRNAs were selected to confirm the expression changes using RT-qPCR analysis, which confirmed the upregulation of connective tissue growth factor (*Ctgf)*, thrombospondin (*Thbs1)*, *serpinE1* and myotrophin (*Mtpn*) and downregulation of solute carrier 2, member 4 (*Slc2A4*) and fibronectin type III domain containing 5 (*Fndc5*) (Figure 3). One mRNA, fibulin 1, was not validated as having a different expression level (not shown), suggesting an overall mRNA validation rate of 86%.

## Discussion

We have determined the miRNA expression levels of early and end-stage HCM in a severe mouse model of the disease. The miRNA expression profiles show a striking resemblance to that of other forms of cardiac stress, and time-course analysis revealed that altered miRNA expression commences at a pre-disease stage. Furthermore, the miRNA signatures can partly explain the differential expression levels of mRNAs at end-stage HCM, and highlight upregulation of genes implicated in cardiac hypertrophy and electrophysiology, calcium signalling, extracellular matrix regulation and the TGF-β signalling pathway as underpinning the development of HCM.

The expression levels of miR-1 and miR-133a were significantly lower at a pre-disease stage in DBL mice, and this represents the earliest recorded pathological change in our well-characterised mouse model of HCM [20]. Downregulation of miR-1 has been reported as early as one day after TAB [8], and while miR-1 is abundant in adult hearts, its level is lower in the developing embryonic hearts of mice [24], [25], suggesting that reversion of miR-1 towards an embryonic expression level is an early response to cardiac stress. The cardiac abundant miR-1 family consist of two copies with identical mature nucleotide sequences, and thus target the same mRNAs [24]. It is unclear what causes the reduction of miR-1 levels in DBL mice, however, serum response factor regulates transcription of both copies in the heart [26]. The heart is sensitive to miR-1 levels, with adenoviral overexpression of miR-1 previously shown to attenuate cardiac hypertrophy, and targeted homozygous deletion of a single copy (miR-1-2) in mice leads to significant embryonic lethality due to defects in cardiogenesis [25], [27]. Furthermore, a requirement for miR-1 during post-embryonic development is suggested by impaired cardiac conduction in the few miR-1-2 homozygous null mice that survive to adulthood, and the high level of miR-1 in the adult heart. The second downregulated miRNA at a pre-disease stage was miR-133a, which belongs to the same transcriptional unit as miR-1 [24]. *In vitro* suppression of miR-133, using an antisense sequence to sequester miR-133, induces hypertrophy, and *in-vivo* inhibition of miR-133 by infusion of an adenoviral antagomir causes cardiac hypertrophy [24]. Together, these data further implicate downregulation of miR-1 and miR-133 in the development of HCM, and strategies to maintain their levels may represent a therapeutic opportunity.

During the early stage of HCM development, more miRNAs were upregulated than downregulated, and this is consistent with the same early response to TAB [6], [7], [9]. This may reflect the challenge of detecting the early stages of miRNA downregulation, particularly for stable mature miRNAs such as miR-208 that has a half-life of >12 days [28]. We confirmed the upregulation of miR-214 and miR-132 during early-stage disease in DBL mice. miR-214 was previously reported as the most strongly upregulated miRNA at end-stage human ischemic- and dilated cardiomyopathy, and following aortic stenosis, and may contribute to cardiac hypertrophy as overexpression in cardiomyocytes induces hypertrophic growth [6], [11]. miR-132 has not been widely implicated in cardiac disease, however, it regulates post-translational expression of the β2-subunit of cardiac L-type calcium channels [29], consistent with changes in electrical conduction in DBL mice.

At age 16 days, the DBL mice progress to an end-stage dilated phase of disease, with marked cardiac fibrosis and myocyte disarray [20]. Consistent with these histopathological changes, there are more differentially expressed miRNAs and with wider differences in expression levels compared to early stage HCM. We detected co-ordinate expression of co-transcribed and clustered miRNAs, which may allow for a combinatorial effect on mRNA regulation. Thus, the downregulation of miR-133 and miR-30 may contribute to the development of cardiac fibrosis in DBL mice, as both regulate the profibrotic signalling factor, *CTGF* [30], which was correspondingly upregulated. Collectively, these data show that distinct miRNAs with roles in cardiac hypertrophy and fibrosis are regulated from a pre-disease stage of HCM and likely play a causal role in regulating the cardiac transcriptome during disease development.

The early and end-stage HCM signature miRNAs include many that are commonly differentially expressed following cardiac stress, irrespective of whether this is from a pathological or physiological stimulus. These include miR-1, miR-133, miR-30 and miR-150 which often show reduced expression, and miR-21, miR-199 and miR-214 which often show increased expression [6], [7], [8], [9], [11], [12], and they may represent miRNAs with a central role in cardiac remodelling. Alternatively, some of these miRNAs may not be directly involved in cardiac pathology, but reflect secondary responses to cardiac remodelling. Irrespective of the cause of cardiac hypertrophy, the downregulation of miR-1 and upregulation of miR-214 seems to be implicated widely in murine cardiac disease, and in human cardiomyopathy. In addition, we validated the increased expression of miR-132 during HCM, which is not widely reported as differentially expressed following cardiac stress and may represent a distinct change in the pathogenesis of severe HCM in DBL mice.

Dysregulated target mRNAs offer an insight into pathways regulated during the onset and development of HCM. Upregulated mRNA targets of miR-1 include myotrophin (*Mtpn*), which can trigger myocardial hypertrophy [31], and *Ctgf* and thrombospondin, which regulate extracellular matrix remodelling, suggesting miRNAs may contribute towards fine tuning of the extracellular matrix proteins [32]. This study did not investigate the effects of miRNA-regulated translation repression, and thus the impact of miRNAs on protein levels is likely to be more extensive. Furthermore, the TLDA cards used in this study represent ∼20% of all known miRNAs, and while these included the most abundant cardiac miRNAs, it is likely that additional miRNAs, and thus target mRNAs, may contribute to the disease development in DBL mice than reported here.

Several lines of evidence indicate that TLDA cards represent a reliable high-throughput screening tool to quantitate miRNA levels. Firstly, TLDA cards utilise RT-qPCR to measure miRNA levels and they have recently been shown to compare favourably to microarray and bead array technologies [33]. Secondly, the 177 cardiac expressed miRNAs with Ct values <32 in DBL and NTG mice correlate well with previous global cardiac miRNA profiles, with some of the discrepancy explained by alternative miRNAs included on different arrays [7], [8], [9]. Thirdly, as previously reported [34], we detected coordinate expression levels of clustered miRNAs, and those co-transcribed, or on opposite arms of a miR-5p:miR-3p stem-loop. Recent advances in RNA analysis using next generation sequencing technologies now provide an unbiased characterisation of both small RNAs and whole transcriptomes in the heart, and may be superior to TLDA arrays for accurately quantifying lower-abundance transcripts [35], [36], [37], [38]. Furthermore, in combination with immunoprecipitation of the cardiac RISC complex and subsequent sequencing of associated mRNAs, it is now possible to identify *in-vivo* miRNA targets [36]. Such methods will allow future studies to identify individual miRNA targets in biological context during disease development.

In conclusion, the current study highlights the regulatory roles of miRNAs in the development of severe HCM in the DBL mouse model. The results define the miRNA expression profiles during development and progression of HCM and highlight gene networks regulated by miRNAs. Understanding the role of miRNA-mRNA interactions will shed light on defining the precise pathogenic mechanisms underpinning HCM, and provide potential platforms for novel diagnostic, disease progression, and therapeutic interventions.

## Supporting Information

Figure S1 **Pairwise Pearson’s product moment correlation coefficient of miRNA Ct values <32 within each group of mice.**
(DOCX)

Figure S2 **Volcano plots of miRNA P-value vs Fold Change at (a) early-stage HCM and (b) late-stage HCM.**
(DOCX)

Figure S3 **Extended time course analysis of miRNA expression levels in A. Upregulated microRNAs, B. Downregulated microRNAs and C. MicroRNAs not validated by RT-PCR.** Fold change is shown compared to NTG mice age 5 days.
(DOCX)

Table S1 (XLSX)
