# Supplementary material for: Global MicroRNA Profiling of the Mouse Ventricles during Development of Severe Hypertrophic Cardiomyopathy and Heart Failure
Source: PLoS One. 2012 Sep 14;7(9):e44744. doi: 10.1371/journal.pone.0044744 (PMC3443088; doi:10.1371/journal.pone.0044744)
Supplement: Figure S1 — Pairwise Pearson’s product moment correlation coefficient of miRNA Ct values <32 within each group of mice. (DOCX) [file pone.0044744.s001.docx]

**Supplementary Figure S1**

Pairwise Pearson’s product moment correlation coefficient of miRNA Ct values <32 within each group of mice.

**
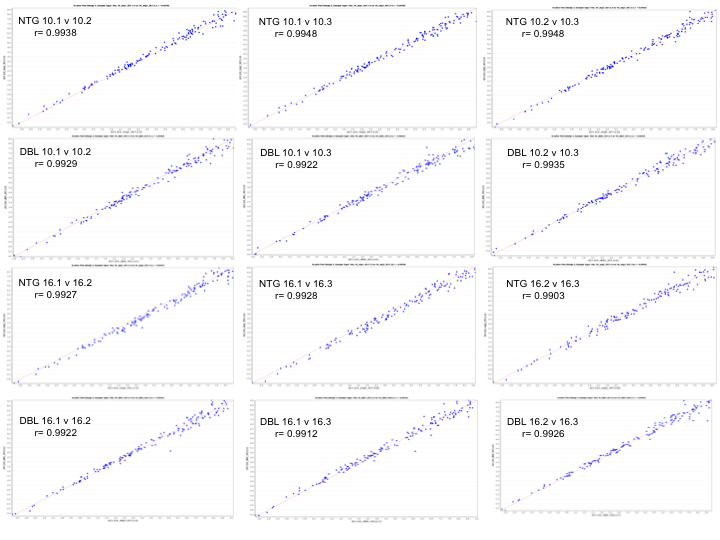
**
