# Supplementary material for: Global MicroRNA Profiling of the Mouse Ventricles during Development of Severe Hypertrophic Cardiomyopathy and Heart Failure
Source: PLoS One. 2012 Sep 14;7(9):e44744. doi: 10.1371/journal.pone.0044744 (PMC3443088; doi:10.1371/journal.pone.0044744)
Supplement: Figure S2 — Volcano plots of miRNA P-value vs Fold Change at (a) early-stage HCM and (b) late-stage HCM. (DOCX) [file pone.0044744.s002.docx]

**Supplementary Figure S2**

Volcano plots of miRNA *P*-value vs Fold Change at **(a)** early-stage HCM and **(b)** late-stage HCM

**
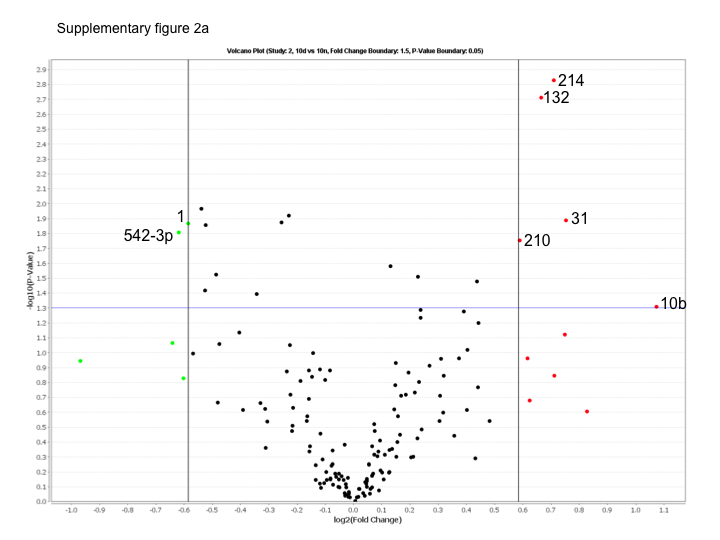
**

**
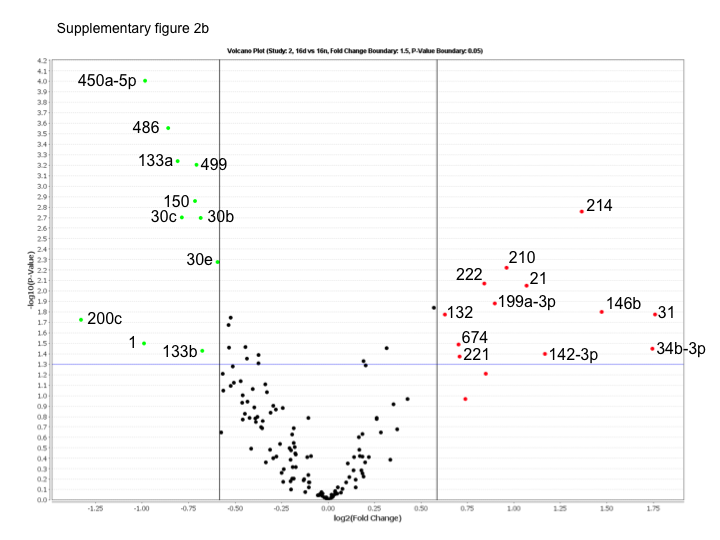
**
