# Supplementary material for: Global MicroRNA Profiling of the Mouse Ventricles during Development of Severe Hypertrophic Cardiomyopathy and Heart Failure
Source: PLoS One. 2012 Sep 14;7(9):e44744. doi: 10.1371/journal.pone.0044744 (PMC3443088; doi:10.1371/journal.pone.0044744)
Supplement: Figure S3 — Extended time course analysis of miRNA expression levels in A. Upregulated microRNAs, B. Downregulated microRNAs and C. MicroRNAs not validated by RT-PCR. Fold change is shown compared to NTG mice age 5 days. (DOCX) [file pone.0044744.s003.docx]

**Supplementary Figure S3.** Extended time course analysis of miRNA expression levels in **A**. Upregulated microRNAs, **B.** Downregulated microRNAs and **C.** MicroRNAs not validated by RT-PCR. Fold change is shown compared to NTG mice age 5 days.


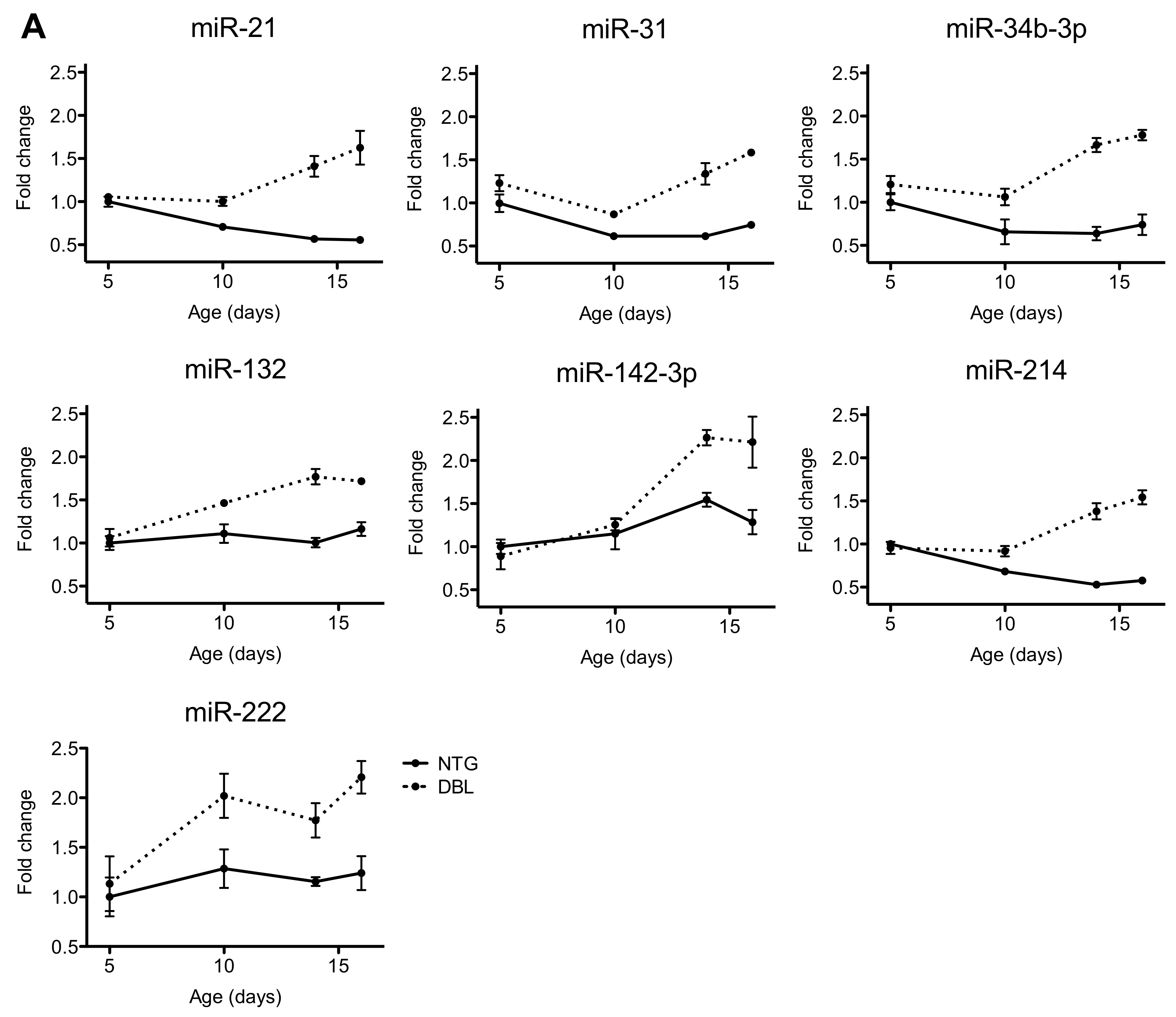


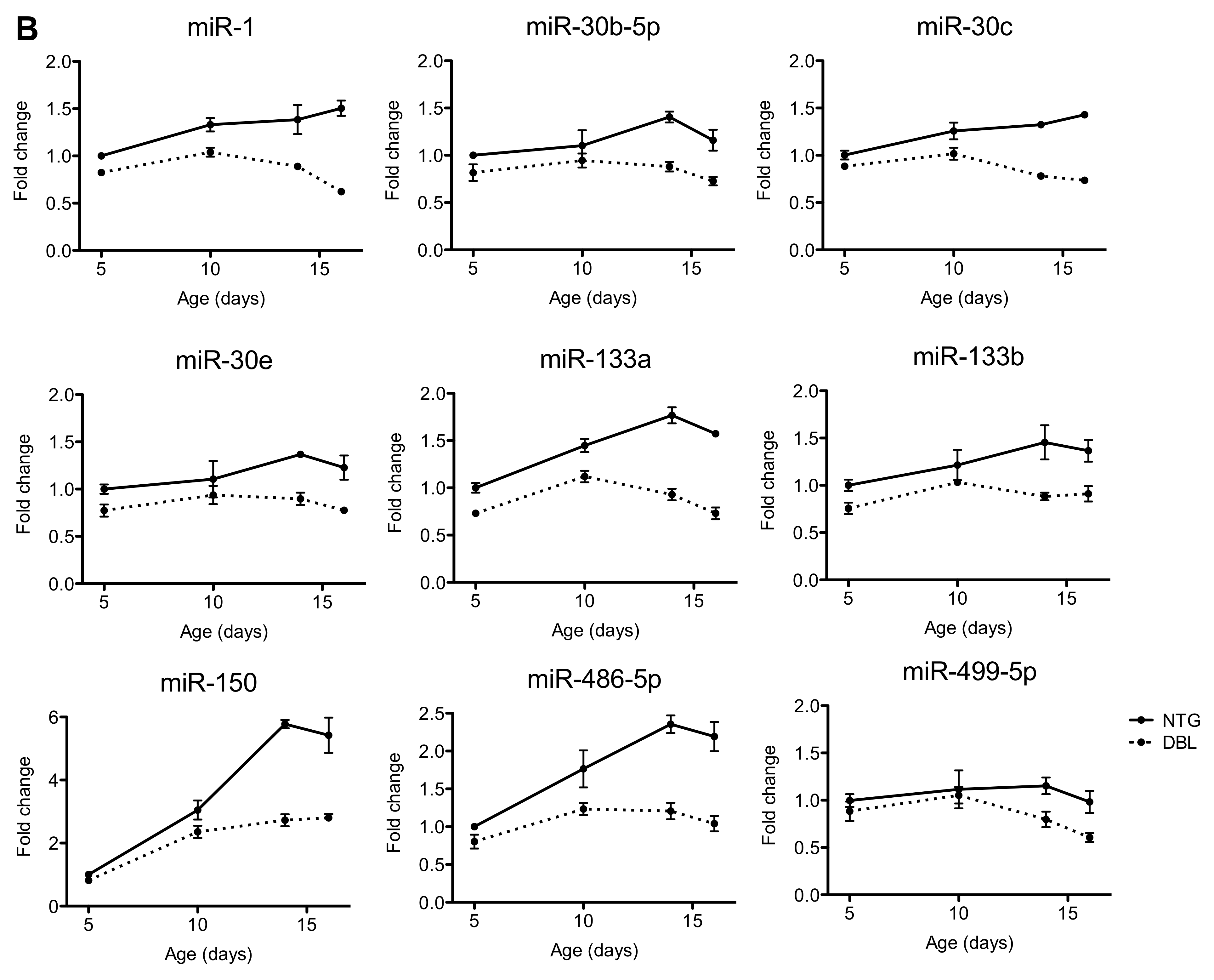


**
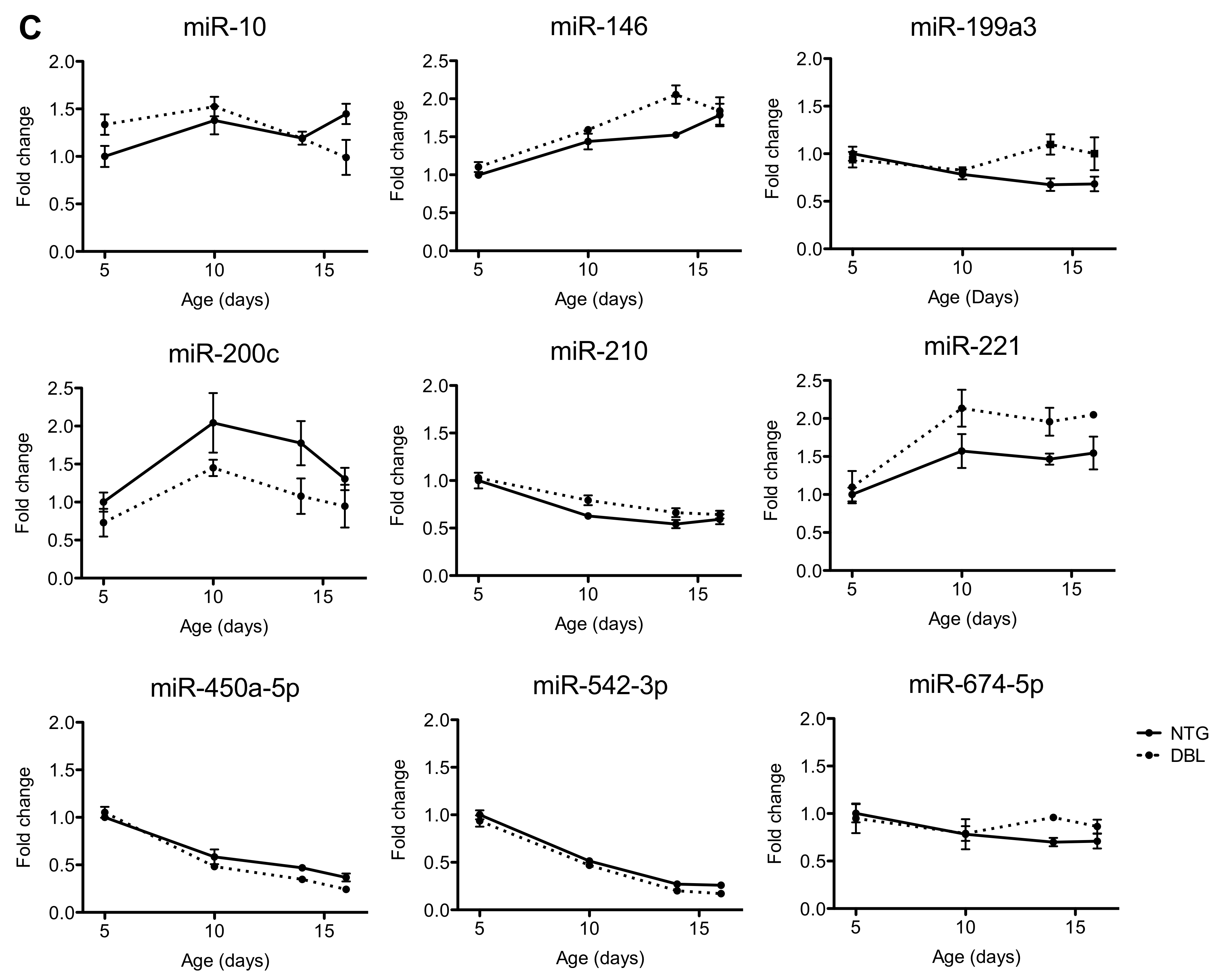
**
